# Improving health literacy through group antenatal care: results from a cluster randomized controlled trial in Ghana

**DOI:** 10.1186/s12884-023-06224-x

**Published:** 2024-01-05

**Authors:** Jody R. Lori, Vida Ami Kukula, Liya Liu, Veronica E.A. Apetorgbor, Bidisha Ghosh, Elizabeth Awini, Nancy Lockhart, Georgina Amankwah, Ruth Zielinski, Cheryl A. Moyer, John Williams

**Affiliations:** 1 Department of Health Behavior and Biological Sciences, School of Nursing, University of Michigan, 400 N. Ingalls Bldg, Ann Arbor, MI United States of America; 2 Dodowa Health Research Centre, Ghana Health Service, P.O.Box DD1, Dodowa, Ghana; 3 Department of Learning Health Sciences, University of Michigan Medical School, University of Michigan, Ann Arbor, MI United States of America

**Keywords:** Group antenatal care, Prenatal care, Group care, Health literacy, Maternal health, Newborn health, Ghana

## Abstract

**Background:**

Although the majority of Ghanaian women receive antenatal care (ANC), many exhibit low health literacy by misinterpreting and incorrectly operationalizing ANC messages, leading to poor maternal and newborn health outcomes. Prior research in low-resource settings has found group antenatal care (G-ANC) feasible for women and providers. This study aims to determine the effect of G-ANC on increasing maternal health literacy. We hypothesized that pregnant women randomized into G-ANC would exhibit a greater increase in maternal health literacy than women in routine, individual ANC.

**Methods:**

A 5-year cluster randomized controlled trial was conducted in 14 rural and peri-urban health facilities in the Eastern Region of Ghana. Facilities were paired based on patient volume and average gestational age at ANC enrollment and then randomized into intervention (G-ANC) vs. control (routine, individual ANC); 1761 pregnant women were recruited. Data collection occurred at baseline (T0) and post-birth (T2) using the Maternal Health Literacy scale, a 12-item composite scale to assess maternal health literacy. Logistic regression compared changes in health literacy from T0 to T2.

**Results:**

Overall, women in both the intervention and control groups improved their health literacy scores over time (*p* < 0.0001). Women in the intervention group scored significantly higher on 3 individual items and on overall composite scores (*p* < 0.0001) and were more likely to attend 8 or more ANC visits.

**Conclusion:**

While health literacy scores improved for all women attending ANC, women randomized into G-ANC exhibited greater improvement in overall health literacy post-birth compared to those receiving routine individual care. Life-saving information provided during ANC must be presented in an understandable format to prevent women and newborns from dying of preventable causes.

**Trial Registry:**

Ethical approval for the study was obtained from the Institutional Review Boards of the University of Michigan (HUM#00161464) and the Ghana Health Service (GHS-ERC: 016/04/19).

## Introduction

Maternal mortality remains a major heath challenge in low- and middle-income countries (LMICs) with an estimated average of 545 maternal deaths per 100,000 live births in 2020 [1]. Even more concerning, sub-Saharan Africa alone accounted for approximately 70% of all maternal deaths in 2020 [1]. Ghana, has an estimated maternal mortality ratio of 263 in 2020 compared to the global ratio of 223 [1]. Additionally, Ghana had a neonatal mortality ratio of 22.9 deaths per 1,000 live births in 2021 [2]. Ghana is one of the 24 priority countries targeted by the United States Agency for International Development (USAID) to improve maternal and child health and end preventable deaths [3].

Antenatal care (ANC) plays a crucial role in the health promotion of pregnant women and their unborn children. As a form of preventive healthcare, women learn about the warning signs during pregnancy and childbirth, understand the implementation of healthy behaviors, and receive support during this critical time [4]. In 2016, Ghana transitioned to the recommended minimum of eight ANC visits during a woman’s pregnancy [5, 6]. This new framework emphasizes the need for comprehensive and patient-centered care at each visit and can reduce perinatal deaths by up to 8 per 1,000 births when compared to the former four visit recommendation [6].

### Antenatal care and health literacy

WHO (1998) defines health literacy as “the cognitive and social skills which determine the motivation and ability of individuals to gain access to, understand, and use information in ways which promote and maintain good health” [7]. Health literacy extends beyond reading or writing and is also associated with positive health behavior, appropriate health service utilization, and acceptance of interventions that maximize health outcomes [8]. More specifically, Renkert and Nutbeam define maternal health literacy as the skills that allow a woman to utilize information they acquire to promote and maintain their health and the health of their children [9].

Studies have shown that women with higher health literacy are more capable of seeking appropriate and timely health care [10]. Conversely, low health literacy is associated with the lack of, or improper use of, healthcare services, increased mortality, and poor self-care [11]. According to Levy and Janke, individuals with low health literacy are significantly more likely to delay care-seeking, even after controlling for other factors including race and ethnicity, poverty, and employment [12].

46% of women in Ghana are reported to have low literacy [13]. (WHO, 2019), placing this subgroup at risk of higher mortality rates related to delayed or lack of care-seeking. In the Eastern District, two-thirds of the population (63.5%) aged 15 years and older are literate, mirroring the country-level statistics [14]. Lori et al. found that many Ghanaian women exhibited low health literacy by misinterpreting and incorrectly utilizing the information presented to them in traditional ANC [15].

A systematic review conducted by Zibellini et al., assessed the effectiveness of health literacy interventions on pregnancy outcomes [16]. Of the 13 studies included in the review, 10 reported on knowledge acquisition and only two reported on health literacy [16]. While several interventions demonstrated the ability to increase health-related knowledge, its impact on maternal health literacy using validated measures could not be evaluated [16]. Increased knowledge alone does not reflect the development of personal and social skills that accompany the contemporary understanding of health literacy, which is more than just knowledge [16, 17].

### Group antenatal care

Numerous studies have shown group antenatal care (G-ANC) as a feasible intervention for pregnant women [18–21]. As an emerging concept, particularly in LMICs, G-ANC is a way of organizing antenatal visits in a group setting and is based on three major components: health assessment, knowledge, and social support [22]. Prior to the start of G-ANC visits, pregnant women are organized into cohorts of 10 to 14 women of similar gestational age. They actively participate in health assessment and discussions led by health-care providers, with the purpose to increase interaction within the group and facilitate peer support during pregnancy [22].

While G-ANC has been conducted in high-resource countries for years, it has only recently been introduced as an alternative to individual antenatal care (I-ANC) in sub-Saharan Africa. Recent studies support G-ANC as an option in resource-constrained countries and populations [18, 23–25]. In addition, evidence has shown that G-ANC results in greater knowledge surrounding nutrition, breastfeeding, changes during pregnancy, and family planning [24]. More specifically, studies have found G-ANC to be acceptable and feasible to both women and providers in Ghana [18, 26]. In a pilot study evaluating the benefits of G-ANC in Ghana, Lori et al. found that G-ANC improved communication and enhanced information-sharing and peer support, providing mothers with the necessary tools to guide behaviors and improve pregnancy outcomes [27]. This study aims to quantify the effect of the G-ANC model in improving maternal health literacy among pregnant women in Ghana using a validated health literacy assessment tool.

## Methods

### Study design and setting

A cluster randomized controlled trial was conducted in 14 facilities in four districts (Nsawam Adoagyiri, Yilo Krobo, Akwapim North, and Lower Manya Krobo) of the Eastern Region of Ghana. Health facilities were randomized using a matched pairs design; each pair was similar in the number of deliveries and average gestational age of the women at enrollment in antenatal care. The trial was registered with clinicaltrials.gov (NCT04033003) and recruitment began 25/07/2019. The locations of the facilities were far enough apart to avoid cross-group contamination. In each pair of facilities, one was randomly assigned to the intervention (G-ANC) and the other to the control (I-ANC). The matching and randomization process was conducted using the nbpMatching package from R software [28]. Sample size was determined by calculating for an intraclass correlation coefficient, which is the extent to which the effect differs across facilities, of 0.01. Due to the nature of the intervention, this was a non-blinded trial of both participants and providers. Full details of the study protocol, including matching, randomization, and power analysis, are available in a prior publication [29]. Recruitment began July 2019 and ended when enrollment targets were met. Data collection ended July 2023 when data collection was complete. Patients and the public were not involved in the design, conduct, reporting or dissemination of the research.

### Intervention

The G-ANC model consisted of nine meetings: one individual meeting and eight group meetings. At the initial ANC visit, women were assigned to small groups consisting of 10 to 14 women of similar gestational age. A systematic synthesis of the literature on G-ANC found most groups ranged in size from 8 to 12 women [17]. To account for potential loss to follow-up, we chose to recruit groups of 10-14 women. Women met individually with the midwife and completed a standard history and physical examination along with laboratory tests. Group visits then started at the second ANC visit. Group visits were led by the midwife who was assisted by a health facility staff member. The midwife, health facility staff member, and patients sat in a circle facing one another for each 60-90 min facilitated, interactive meeting. Strategies used included storytelling, peer support, demonstration, role-plays and teach-back. Picture cards were used to enhance communication and learning in the group setting, providing a mechanism to envision new concepts and ideas. Examples of topics include the use of bed nets and malaria prophylaxis, saving money for transportation, identifying danger signs, positions for birth, family planning options, and caring for a newborn. Women were encouraged to discuss and share the information with family members and friends, reinforcing what was learned during ANC visits. Content was repeated multiple times in a variety of ways to enhance retention, including auditory (listening to stories and signs of problems), visual (through demonstration and picture cards), kinesthetically (practicing actions and ‘handling’ picture cards), and the “Take Action Card” Booklet for home use. The booklets, comprised of pictures corresponding to each topic covered in the group sessions, were provided for each pregnant woman to take home and use as a reminder of actions to take if problems arose.

### Recruitment of participants and informed consent

Recruitment of women occurred at individual health facilities, with research staff working closely with clinic staff to identify women attending their first ANC visit and who met the eligibility criteria. The criteria were as follows: (1) less than 20 weeks gestation, (2) speaks Dangme, Ga, Akan, Ewe, or English, (3) over the age of 15, and (4) not considered high risk. Women meeting the eligibility criteria and who indicated an interest in learning more about the study to the midwife were instructed to talk to the research assistant (RA). The RA explained the study, and those women willing to participate were taken through an informed consent procedure.

Ethical approval for the study, including the informed consent process, was obtained from the Institutional Review Boards of the University of Michigan (HUM#00161464) and the Ghana Health Service (GHS-ERC: 016/04/19). Written informed consent was obtained individually from all participants by the RA and witnessed by a second person. The informed consent procedure was conducted in private with a witness (health facility staff) present. The written informed consent was given to each potential participant. Due to the general low-literacy rate in Ghana, the RA read the consent form aloud to each individual. A teach-back method was used to confirm understanding of the research purpose, benefits, risks, and procedures, and methods and questions were invited until the information was clear. Women who agreed to participate signed the informed consent form and the witness signed that he/she was present during the informed consent process, that all questions were answered, and that women voluntarily agreed to take part in the research. Finally, the informed consent form was signed by the RA and a copy given to the participant. As pregnant minors age 15–18 are emancipated, the informed consent process (no requirement for parental consent) was approved by University of Michigan IRB as well as Ghana Health Service for participants under the age of 18.

### Measures

Multiple measures were used for data collection. The Maternal Health Literacy (MaHeLi) scale served as the fundamental tool to assess the health literacy of participants in this study. Developed and tested with 384 adolescents attending ANC in Uganda, the 12-item version of the MaHeLi scale was applied and validated for use, [29] from the original 20-item scale. The modified version of the MaHeLi scale consists of 12 yes-or-no questions to assess maternal health literacy among the participants. The scale primarily assesses three pertinent aspects of health literacy: health-seeking behavior (HSB), competence and coping skills (CCS), and the appraisal of health information (AHI) [30, 31].

Health seeking behavior is defined as actions taken by an individual who perceives themselves to be ill to find appropriate treatment [30]. Competence and coping skills refers to the self-evaluative judgements that mothers have about their ability to take and accomplish the tasks in pursuit of adequate health status for themselves and their children [30]. Appraisal of health information demonstrates the cognitive and literacy skills required to understand and interpret health information that is provided and perceived. Cognitive skills include comprehension, analysis, synthesis, and evaluation, all of which enable understanding of the relevance and application of information [30].

### Data collection and statistical analyses

Baseline demographic data were collected upon enrollment in the study following informed consent. Health literacy data were collected at two time points: baseline data (T0), which was collected immediately after the informed consent process prior to the start of ANC, and post-birth (T2) 6–12 weeks post-birth using the modified MaHeLi scale. All data were collected by trained RAs using encrypted and password-protected tablets, and entered into REDCap, a secure web-based application for data collection and database management [32, 33].

Data management was done using SAS 9.4 while data analysis was conducted using Stata 17.0. Results from T0 and T2 were compared between intervention and control groups to assess the efficacy of G-ANC on improving maternal health literacy compared to I-ANC. Logistic regression adjusted for clustering was used to analyze changes in maternal health literacy from T0 to T2 and between the control and the intervention groups for the 12 yes/no items corresponding to maternal health literacy. The following covariates were considered for each of the analyses: (1) time, study arm, and time*study arm; (2) age (continuous); (3) first pregnancy (1 = yes/0 = no); (4) marital status (single/separated/divorced, married/living together, in a relationship with financial support); (5) highest level of education (primary, middle/JHS/JSS, secondary/SHS/Technical/Vocational, and tertiary; and 6) who typically has the strongest voice in deciding when and where you seek healthcare (1 = self, 0 = others). Bonferroni correction was made where applicable for multiple comparisons for all models, a reduced model consisting of significant effects were obtained via backward selection method. Chi-square was used to compare attendance at 8 or more ANC visits by women enrolled in G-ANC vs. women receiving I-ANC 8 or more ANC visits.

## Results

### Demographics

A total of 1761 participants (877 in G-ANC and 844 in I-ANC) were recruited into 120 groups of 10 to 14 women of the same gestational age range upon presentation for ANC and completed data collection at enrollment. Of the 1761, 260 were either lost to follow up or did not attend G-ANC in the intervention arm and 216 were lost to follow up in the control group. Data analysis included only participants assigned to their original group. The majority of participants were less than 35 years old (84%), and most were either married, cohabitating, or living together with their significant other (96%). Only 20% of participants were pregnant with their first child (Table 1). There were no significant differences between the intervention and control groups with the exception that more women were single, divorced or widowed in the control group (*p* < 0.0001).

**Table 1:** Demographics of the participant population

Categorical Variables	Overall (N = 1761*)	Control (n = 884)	Intervention (n = 877)	*p* value
**Age**				
< 25	501 (28%)	266 (53%)	235 (47%)	0.1930
25–34	987 (56%)	477 (48%)	510 (52%)	
35 or older	273 (16%)	141 (52%)	132 (48%)	
**Relationship**				
Single/Divorced/Widowed	70 (4%)	53 (76%)	17 (24%)	< 0.0001
Married/Cohabitating/Living Together	1691 (96%)	831 (49%)	860 (51%)	
**Maternal Education**				
Primary	246 (14%)	120 (49%)	126 (51%)	0.6895
Middle/JHS^a^/JSS^b^	829 (49%)	429 (52%)	400 (48%)	
Secondary/SHS^c^/Technical/ Vocational	459 (27%)	223 (49%)	236 (51%)	
Tertiary	164 (10%)	83 (51%)	81 (49%)	
**Partner Education**				
Middle/JHS^a^/JSS^b^ or less	666 (39%)	335 (50%)	331 (50%)	0.8525
Secondary	627 (37%)	306 (49%)	321 (51%)	
Tertiary	261 (16%)	126 (48%)	135 (52%)	
N/A, Unknown	137 (8%)	64 (47%)	73 (53%)	
**Religion**				
Christianity	1646 (93%)	835 (50.4%)	811 (49.6%)	0.1185
Muslim	97 (6%)	39 (39%)	58 (61%)	
Other	18 (1%)	10 (56%)	8 (44%)	
**First Pregnancy**				
No	1412 (80%)	703 (50%)	709 (50%)	0.4876
Yes	349 (20%)	181 (52%)	168 (48%)	
**Location of Delivery**				
Hospital/Polyclinic/ Health Center	1711 (97%)	853 (50%)	858 (50%)	0.0904
Other	50 (3%)	31 (62%)	19 (38%)	
**Continuous Variables** **Mean (SD)**				
Maternal age	28.2 (5.8)	28.1 (6)	28.3 (5.6)	0.5042
Wealth index	6.8 (2.4)	6.9 (2.4)	6.9 (2.3)	0.6174
**Number of previous pregnancies**	3.5 (1.4)	3.5 (1.4)	3.5 (1.5)	0.7075

^*^Total sample size. Cells that do not add across represent missing data

Categorical variables were compared using chi-square test

Maternal age and wealth index tested using 2-sample t-test

Number of previous pregnancies tested using Mann Whitney Wilcoxon test (non-parametric)

^a^JHS = junior high school. ^b^JSS = junior secondary school. ^c^SHS = senior secondary school

### Health literacy

Overall, women in both I-ANC and G-ANC improved their composite health literacy scores between baseline and post-birth (*p* < 0.0001). Women in the control group increased their score from 9.7 at T0 to 11.0 at T2. However, women in the intervention group improved their health literacy scores significantly more than the control group with an increase from 9.6 at T0 to 11.3 at T2 out of a possible 12 points (*p* < 0.0001).

The change in scores for women in intervention groups were significantly different on three individual items (Table 2) compared to the control groups over time. The ability to utilize cognitive skills and reason with health information and symptoms significantly increased among women receiving G-ANC, as shown in responses to “Can you tell the difference between myths and truths about pregnancy related information?”. Perceived social support also significantly increased among women receiving group care compared to those receiving individual care as noted in response to “Can you comfortably rely your health concerns to people around you?” and “Have you remained active in social gatherings?”. Education was found to be a significant predictor for these three items. With the reference group being the primary educated women, the odds ratios (OR) for each of the 3 items are summarized. For the question about being able to differentiate between myths and truths about pregnancy, the OR for middle, secondary and tertiary school educated women are 1.40 (CI:1.1, 1.9), 2.16 (CI: 1.6, 3.0) and 5.23 (CI: 3.3, 8.3). The OR for the perceived social support question for middle, secondary and tertiary educated women are 1.37 (CI: 1.0,1.9), 2.19 (CI: 1.4, 3.4) and 2.72 (CI: 1.5, 5.0). For the final question about remaining active in social gatherings, the OR for middle, secondary and tertiary educated women are 0.98 (CI: 0.8, 1.3), 1.40 (CI: 1.1, 1.8) and 1.67 (CI: 1.2, 2.4). Results from the final model consisting of the significant covariates are presented in Table 2. The odds-ratio estimates for all the predictors are presented in the table.

**Table 2:** Significant covariates for MaHeLi scale

Item/Question	Can you tell the difference between myths and truths about pregnancy related information? CCS^a^		Can you comfortably relay your health concerns to people around you? AHI^b^		Have you remained active in social gatherings? AHI^b^	
Predictors	Odds Ratio (95% CI)	*P*-value	Odds Ratio (95% CI)	*P*-value	Odds Ratio (95% CI)	*P*-value
**Study arm (Ref: Control)**						
Intervention	0.97 (0.8, 1.2)	0.7640	1.65 (1.3, 2.1)	< 0.0001	1.05 (0.8, 1.3)	0.6770
**Time (Ref: Time = 0)**						
Time = 2	2.45 (1.9, 3.1)	< 0.0001	2.97 (2.1, 4.1)	< 0.0001	4.99 (4.0, 6.2)	< 0.0001
Study arm*Time (Ref: Control, time = 0)						
Intervention, time = 2	3.56 (2.3, 5.5)	< 0.0001	3.09 (1.4, 6.7)	0.0040	1.73 (1.2, 2.4)	0.0010
**Education (Ref: Primary)**						
Middle	1.40 (1.1, 1.9)	0.0170	1.37(1.0, 1.9)	0.0780	0.98 (0.8, 1.3)	0.9830
Secondary	2.16 (1.6, 3.0)	< 0.0001	2.19 (1.4, 3.4)	< 0.0001	1.40 (1.1, 1.8)	0.0150
Tertiary	5.23 (3.3, 8.3)	< 0.0001	2.72 (1.5, 5.0)	0.0020	1.67 (1.2, 2.4)	0.0070
**First pregnancy (Ref: No)**						
First pregnancy (yes)	0.70 (0.5, 0.9)	0.0050	0.66 (0.5, 0.9)	0.0160	N/A	
**Strongest voice (ref: Others)**						
Self	N/A		N/A		1.56 (1.3, 1.9)	< 0.0001
**Age**	1.04 (1.02,1.06)	< 0.0001	N/A		N/A	

In addition to enrollment in G-ANC as a significant predictor of improved health literacy, education was found to be statistically significant in improving health literacy scores across groups. The regression table for the MaHeLi scale is reported in Table 3. Women with less education, in general, were more likely to improve their scores more from time 0 to 2. Women with a primary education had a slightly higher increase in health literacy scores when compared to women with a secondary education (difference: 0.44, 95% confidence interval [95% CI]: 0.05–0.83) or tertiary education (difference: 0.77, 95% CI: 0.3–1.25). Increase in the health literacy scores for women with a middle school education is greater than the corresponding increase in women with a tertiary education (difference: 0.50, 95% CI: 0.1–0.9). Finally, increase in the health literacy scores for women with a secondary school education is greater than women with a tertiary education (difference: 0.33, 95% CI: 0.09–0.75).

**Table 3:** Educational status as a predictor of health literacy

Score Difference (T2-T0)	Coefficient	Std Error	*p*-value	95% CI
**Study Arm**				
Intervention	0.44	0.09	< 0.0001	0.26,0.61
**Education**				
Middle	-0.28	0.14	0.0410	-0.55,-0.01
Secondary	-0.44	0.15	0.0030	-0.73,-0.15
Tertiary	-0.77	0.18	< 0.0001	-1.13,-0.42

### Attendance at ANC visits

Table 4 shows that women in the intervention group were significantly more likely to attend the recommended 8 or more ANC visits than women in the control group 81.9% vs. 64.6% (*p* < 0.0001).

**Table 4:** Comparing number of ANC visits by group

	Less than 8 ANC visits n (%)	8 or more ANC visits n (%)	Total N
Individual ANC	186 (35.4%)	340 (64.6%)	526
Group ANC	101 (18.1%)	456 (81.9%)*	557
Total	287	796	1083**

* *p*-value < 0.0001

**Missing = 678, lost to follow-up, withdrawn, or data not recorded in data source

## Discussion

A strength of this study is the randomization design, promoting comparability of the study groups. The similarity between the intervention and control groups, as noted in Table 1, found the only significant difference between group members with the exception that more women were single, divorced or widowed in the control group (*p* < 0.0001), allowing for statistical inferences on the intervention group [34]. The effectiveness of ANC depends on the multidimensional concept of health literacy. Initially considered only as a patient’s ability to read and understand written information, it is now more broadly defined as a person’s ability to acquire or access information, understand it, and use the information in ways that promote and maintain good health [8, 9]. Health literacy scores improved for all women attending ANC; however, women randomized into G-ANC exhibited greater improvement in overall health literacy post-birth compared to those receiving routine individual care.

Although ANC visits provide pregnant women with an abundance of health information, women must develop and utilize cognitive skills to comprehend the information and take appropriate behavioral actions that promote their health and that of their unborn child [9]. Women receiving G-ANC improved significantly on one item in the cognitive domain of reasoning, “Can you tell the difference between myths and truths about pregnancy related information?” compared to their I-ANC counterparts. This finding suggests that G-ANC among pregnant women with low health literacy enhances the cognitive skills necessary to evaluate, distinguish, and reason through information. The improvement of maternal health literacy cognitive skills provides pregnant women the potential to make behavioral decisions and take actions that improve health outcomes and reduce risks for themselves and their children.

Additionally, perceived social support significantly improved in G-ANC compared to those receiving standard I-ANC. Interventions based on social support are effective in promoting healthy maternal prenatal care behaviors [35]. Through providing ANC in a group format, pregnant women can create friendly relationships with one another and share information, advice, and experiences [27]. Social support may increase self-efficacy among these pregnant mothers, [27] which is linked to an increase in healthy behaviors [35]. Studies have shown that social support helps buffer the negative impacts of stressors on health, thus promoting healthier outcomes [36]. Pregnant women who can comfortably relay their health concerns to those around them may allow others to share and alleviate the women’s burdens during the prenatal period. Additionally, by remaining active in social gatherings, pregnant women may strengthen their connection with the community and reap the benefits of a social support system [37].

For a positive pregnancy experience and to improve maternal and newborn outcomes, WHO recommends 8 or more ANC visits [6]. Recent evidence from Ghana found that most women do not meet the current recommendations of ANC with only 31.2-41.9% attending eight ANC visits [38, 39]. Women receiving G-ANC were significantly more likely to comply with this new recommendation than women attending I-ANC, far exceeding the reported range of attendance. Findings from this study found that improving health literacy supports the use of appropriate health service utilization.

### Limitations

This study is limited to pregnant women within the Eastern Region of Ghana. Findings from this study may not necessarily apply to the greater population of pregnant women. Data for those who were lost to follow up was not available so we do not know whether they moved, miscarried or if there were other reasons for discontinuation. While our findings were statistically significant, additional research is needed to determine if G-ANC improves birth outcomes. More research may be needed in other LMICs to determine whether G-ANC is a feasible intervention to implement and whether it improves maternal health literacy in those communities. Another limitation of this study is the lack of repetition of the measure beyond 6 months; therefore, no analysis is available regarding the long-term retention of information and skills acquired by the women during the ANC visits.

Although these limitations exist, this study utilized a strong study design; the cluster randomized controlled trial presented statistically significant improvements in health literacy among low-literate pregnant women who received G-ANC in Eastern Ghana. Additionally, previous studies of health literacy interventions on pregnancy outcomes have only assessed health literacy at a single timepoint [16]. Women in this study were assessed at two timepoints: baseline (prior to the start of ANC) and at 6–12 weeks post-birth.

## Conclusion

Low health literacy among pregnant women in Ghana has contributed to high maternal and neonatal mortality rates due to preventable causes. Therefore, life-saving information provided during ANC must be presented in a format that enables women to understand and use the information to promote positive health behaviors, appropriately use health services, and maintain good health. Improving maternal health literacy through G-ANC may empower women to use newly acquired cognitive skills and take advantage of the established social support to advocate, maintain, and promote their health and the health of their children.

## Data Availability

The datasets generated and/or analyzed during the current study are available in the Deep Blue repository: https://deepblue.lib.umich.edu/data/concern/data_sets/zg64tm83q?locale=en and https://deepblue.lib.umich.edu/data/concern/data_sets/qv33rx26p?locale=en.

## Abbreviations

ANC: Antenatal Care
G-ANC: Group Antenatal Care
I-ANC: Individual Antenatal Care
LMIC: Low- and middle-income countries
MaHeLi: Maternal Health Literacy Scale
HSB: Health-seeking behavior
CCS: Competence and Coping Skills
AHI: Appraisal of health information
WHO: World Health Organization
